# Insight on the deterioration of cultural objects: a multi-analytical approach to characterize degradation products of *lead weights* from a *Steinway & sons* piano

**DOI:** 10.1007/s11356-023-29790-1

**Published:** 2023-09-14

**Authors:** Antonio Faggiano, Concetta Pironti, Oriana Motta, Ylenia Miele, Antonino Fiorentino, Nadia Marchettini, Maria Ricciardi, Antonio Proto

**Affiliations:** 1 Department of Chemistry and Biology, University of Salerno, via Giovanni Paolo II 132, 84084 Fisciano, (SA), Salerno Italy; 2 Consorzio Interuniversitario per la Scienza e la Tecnologia dei Materiali (INSTM), 50121 Firenze, Italy; 3 Department of Medicine Surgery and Dentistry, University of Salerno, via S. Allende, 84081 Baronissi, (SA), Salerno Italy; 4 Department of Earth, Environmental and Physical Sciences, University of Siena, Pian dei Mantellini 44, 53100 Siena, Italy

**Keywords:** Piano, Lead, Acetic acid, Formic acid, Degradation

## Abstract

The identification of the degradation products in objects of cultural significance, including musical instruments (e.g., a piano), is a key issue for the preservation and valorisation processes of cultural heritage. The aim of this study is to characterize the degradation products of *lead weights* from an important *Steinway & sons* piano using a multi-analytical approach that includes ionic chromatography (IC), X-ray diffraction (XRD) and Fourier transform-infrared (FTIR) spectroscopy analyses. These techniques allowed us to identify hydrocerussite as the main degradation product on the superficial layer of *lead weights*, followed by lead acetate and formate. Moreover, accelerated corrosion experiments in closed environments were performed under acetic and formic acid atmospheres to evaluate the development of lead acetate and formate over time. Exposure of *lead weights* to formic and acetic acid vapours leads to the prevalent formation of basic lead formate, which promotes the formation of hydrocerussite. These results can help to limit the degradation of these piano components and consequently preserve the sound of the piano itself.

**Supplementary Information:**

The online version contains supplementary material available at 10.1007/s11356-023-29790-1.

## Introduction

Musical instruments constitute a specific type of cultural heritage and require special care from experts of various disciplines, such as conservators, restorers and scientists (Vaiedelich and Fritz 2017). To study the degradation phenomena of the materials that make up the cultural object turns out to be essential for preserving its unique characteristics. To limit their extent, it is necessary to consider what environmental conditions favour these phenomena. Also, particularly important is the relationship existing between the constituent materials and the sounds developed, especially in the case of violins, that are among the most investigated musical instruments (Fichera et al. 2018; Invernizzi et al. 2020). Regarding the piano, an instrument of choice in classical music (Bokiau et al. 2017), in addition to the preservation of its constituent wood, other elements are crucial in the restoration operations: these are the so called *lead weights* (LW) that constitute a fundamental part of piano keys, since they enable its sound (Costa and Urban 2005; Watkinson 2010). Moreover, the nature of the materials of which the piano is made and the environmental conditions to which it is exposed must also be considered (Butlin 1990; Chiavari et al. 2008; Fichera et al. 2018).

Microclimatic conditions and indoor pollutants are often responsible for some degradation phenomena of works of art (Slezakova et al. 2011; Kontozova-Deutsch et al. 2011b; Chiantore and Poli 2021; Motta et al. 2022; Pironti et al. 2022). In addition, building materials can act as sources of indoor pollutants affecting the work of art itself. Air pollutants that are typically monitored at cultural heritage sites, such as museum environments and religious sites (churches, sanctuaries), include sulphur, nitrogen and carbon oxides, but also ozone, ammonia and particulate matter (Krupińska et al. 2012; Griesser et al. 2016; Pironti et al. 2023). In fact, high concentrations of these compounds are responsible for the degradation phenomena of the artifacts exposed to them. Carboxylic acids, such as acetic acid (AcOH) and formic acid (FOH), are considered dangerous for some works of art, even at low concentrations (μg/m^3^), so they pose a major problem for the conservation in museums (Grzywacz and Tennent 1994). Limestone, ceramics, bronze, lead and copper undergo irreversible damage due to the presence of these organic acids (Tétreault et al. 2003; Hodgkins et al. 2011), and, over time, this phenomenon may cause the complete deterioration of the artifacts containing these materials. The source of AcOH and FOH is well known and mostly includes wood and wooden products (Kontozova-Deutsch et al. 2008; Alapieti et al. 2020). As a consequence, several studies are reported in the literature concerning sampling and analytical methodologies for AcOH and FOH detection in the field of cultural heritage (Johnson et al. 1994; Kontozova-Deutsch et al. 2008, 2011a; Krata et al. 2009; Degano and La Nasa 2016; Smedemark et al. 2020; Kraševec et al. 2021; Michalski et al. 2021).

Concerning the atmospheric degradation of lead, generally lead oxide (PbO) is produced first; subsequently plumbonacrite (Pb_5_O(OH)_2_(CO_3_)_2_) and hydrocerussite (Pb_3_(OH)_2_(CO_3_)_2_) are formed by reaction with carbon dioxide (Niklasson et al. 2008; Inberg et al. 2018). AcOH and FOH are very reactive with lead, so the LW can be easily degraded due to the exposure to these acids emitted from the wood from which the piano is made (Graedel 1994; Lyon 2010; Deflorian and Fedel 2013; Eggert and Fischer 2021). Literature studies suggest that such acid compounds can accelerate the lead corrosion and degradation of lead artifacts by promoting the formation of cerussite (PbCO_3_), plumbonacrite and hydrocerussite (Graedel 1994; Tétreault et al. 1998).

Recently, we analysed for the first time the degradation compounds of one LW sample from a *Steinway & sons* piano (Faggiano et al. 2022). These preliminary results suggested that the lead of LW degraded into hydrocerussite. In this study, we extended the number of samples analysed in order to confirm this interesting finding and completely characterize these compounds using a multi-analytical approach. Furthermore, we decided to perform accelerated corrosion experiments under AcOH and FOH atmospheres to evaluate the development of the degradation products over time.

## Materials and methods

### Materials

All the chemicals used for the measurements (NaOH solution 49–51%, CH_3_CO_2_Na, HCO_2_Na, NaNO_3_, NaCl, Na_2_SO_4_, Acetic acid 96%, Formic acid, KBr, Mg(NO_3_)_2_) were purchased from Sigma-Aldrich (St. Louis, MO, USA). LW samples (6 not degraded and 4 degraded) from a *Steinway & sons* piano (manufactured in 1953 and made of Bavarian spruce wood) were provided by a private restorer.

### Samples exposed to formic and acetic acid vapours

FOH and AcOH atmospheres were prepared by adding 1 mL of both acids to 50 mL of saturated solution of Mg(NO_3_)_2_ at the bottom of the 750 cm^3^ desiccator, as previously reported (Gibson and Watt 2010). The use of saturated solution of Mg(NO_3_)_2_ allows us to obtain a value of relative humidity (RH) of about 54%, which is typical of museum environments. The atmospheric concentrations of FOH and AcOH were determined experimentally using passive samplers after 1 week. Prior to acid vapours exposure, non-degraded samples were sonicated in distilled water and the IC analysis of the washings confirmed the absence of soluble anions on LW surface. Four non-degraded samples (one per exposure time) were placed in the mixed acids environment. As before the start weight, *m*_*0*_, after the specific time of exposure (1, 2, 3 and 4 weeks), the sample was removed, equilibrated in a desiccator at 25 °C for 2 h, and finally weighed. The weight increment (M) at the different exposure times was calculated using the following equation:

1 $${M}_i=\left({m}_i-{m}_0\right)/{m}_0\ast 100$$

where *m*_*i*_ is the mass (g) of LW after the specific time of exposure (*i* = 1, 2, 3 and 4 weeks) and *m*_*0*_ is the mass (g) of LW before the exposure to organic acid vapours. Similarly, the weight increment of formate (F) and acetate (A) was calculated using Eqs. 2 and 3.

2 $${F}_i={f}_i/{m}_0\ast 100$$

3 $${A}_i={a}_i/{m}_0\ast 100$$

where *f*_*i*_ and *a*_*i*_ are the mass (g) of formate and acetate ions, respectively, obtained from ion chromatography measurements, after the specific time of exposure (*i* = 1, 2, 3 and 4 weeks) and *m*_*0*_ is the mass (g) of LW before the exposure to organic acid vapours.

### Passive sampling

Palmes tubes were purchased by Gradko Ltd., UK, and used to evaluate the atmospheric concentration of FOH and AcOH in the prepared mixed acids environment. Samplers were prepared by pipetting an exact volume (40 μL) of the sorbent solution (1 M of KOH and 10% v/v of glycerol) into two stainless steel mesh discs placed inside one cap of the tube and stored in the refrigerator before use (Gibson et al. 1997a, b; Malagodi et al. 2017). After sampling, 3 mL of distilled water was used to extract the trapped analytes on the stainless-steel mesh discs. The concentration of acetate and formate ions present in the extracts was determined by ion chromatography and used to obtain AcOH and FOH masses sampled. The values were converted into air concentrations, expressed as mg/m^3^, using the procedure reported in the supporting material.

### Sample treatment and analytical determinations

To recover the degradation patina on LW, samples were placed into test tubes together with 6 mL of distilled water and put into an ultrasonic bath for 1 h. Subsequently, the obtained suspensions were divided into two parts: one was filtered on 0.45 μm filter and stored for ion chromatography analyses (isocratic hydroxide selective anion-exchange column and conductivity system detector (Ricciardi et al. 2022)); the other was slowly evaporated on a glass slide to perform directly X-ray diffraction measurements (Ricciardi et al. 2018), thereafter, the solid was removed from the glass slide and mixed with potassium bromide to prepare the disk for infrared spectroscopy analyses (Della Monica et al. 2019). In order to achieve proper separation of formate and acetate ions peaks, several experimental conditions were tested: sodium hydroxide eluent concentration from 1 to 20 mM, suppressor current from 20 to 60 mA and flow rate from 0.8 to 1.2 mL/min. The best results were achieved using a sodium hydroxide concentration of 5.0 ± 0.3 mM, a suppressor current of 50 mA and a flow rate of 1.0 mL/min. Under these optimized conditions, we obtain the following parameters for the two carboxylates:

- Acetate: regression line *y* = 0.06286 x + 0.00330; *R*^2^ = 0.99905; LOD: 0.28 mg/L; LOQ: 0.86 mg/L;
- Formate: regression line *y* = 0.16146 x − 0.02249; *R*^2^ = 0.99993; LOD: 0.12 mg/L; LOQ: 0.35 mg/L.

For all the instrumental details, see supporting materials.

## Results and discussion

### Analyses of degradation products on LW samples

Degraded LW samples from a *Steinway & sons* piano were characterized in terms of composition of the degraded layer using different analytic techniques: IC, XRD and FTIR. The last two were performed on the patina removed by sonication in water and dried on a glass slide in order to identify the overall deterioration products formed on LW.

**Fig. 1:**
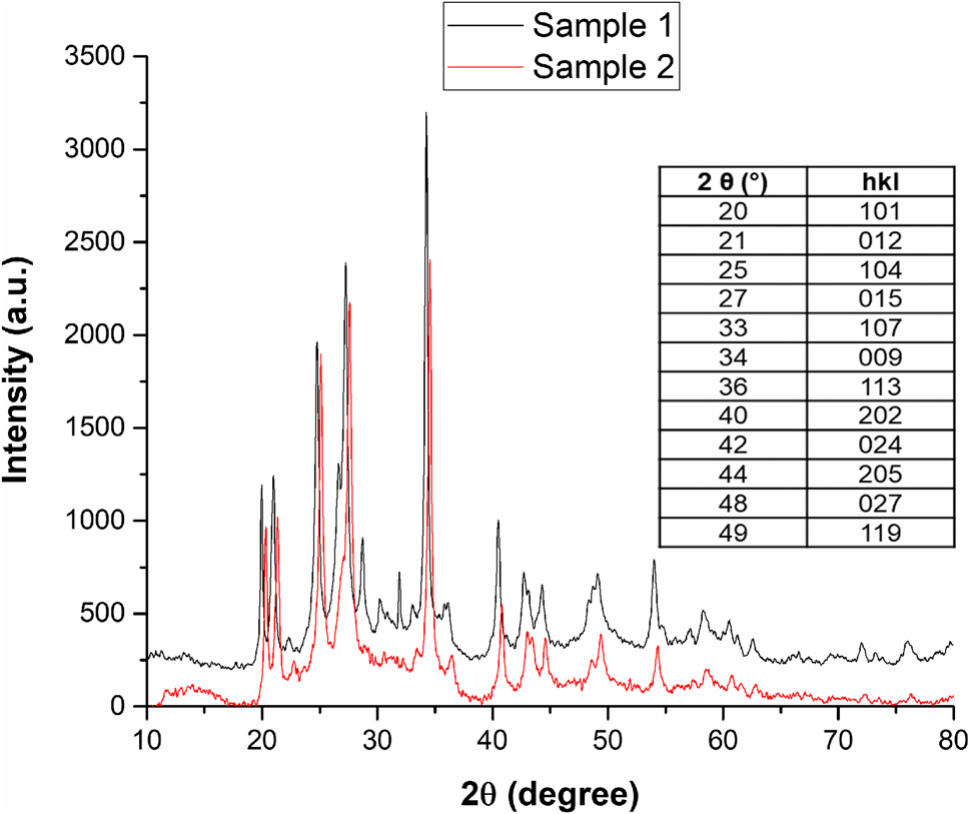
XRD spectra of two degraded samples of LW with the assignment of hydrocerussite peaks

**Fig. 2:**
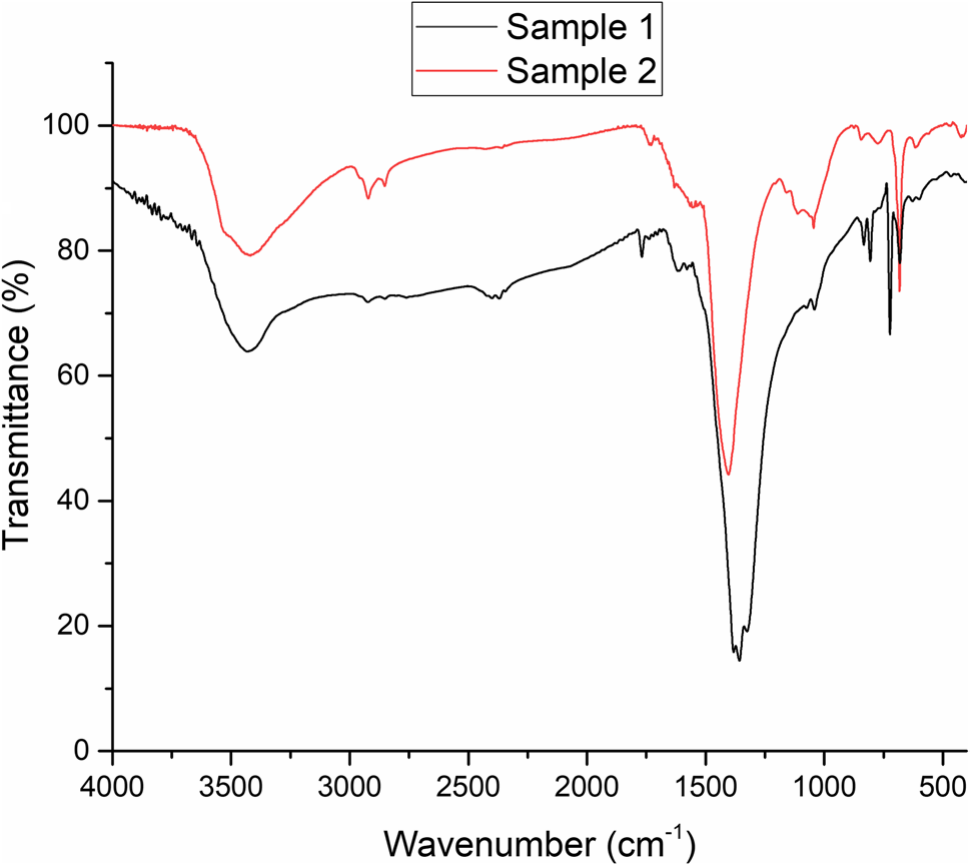
FTIR spectra of two degraded samples of LW

X-ray diffraction spectra of degraded samples (Fig. 1) show the typical diffraction pattern of the trigonal (*R*$\overline{3}$*m*) structure of hydrocerussite, Pb_3_(OH)_2_(CO_3_)_2_, as identified by Miller indices. FTIR spectra (Fig. 2) confirmed the presence of hydrocerussite as main component of degraded patina on lead artifacts, with the following characteristic signals: O—H stretching vibrations band (3500 cm^−1^), ν_3_ asymmetric C—O stretching vibrations of CO_3_^2−^ anions (1400 cm^−1^), ν_1_ symmetric C—O stretching vibrations of CO_3_^2−^anions (1045 cm^−1^), ν_2_ out-pf-plane bending vibrations of CO_3_^2−^anions (835 and 805 cm^−1^) and ν_4_ in plane bending vibrations of CO_3_^2−^anions (684 cm^−1^) (Brooker et al. 1983; Siidra et al. 2018).

The determination of water-soluble compounds on degraded samples was performed by anionic chromatography on the patina removed by sonication in water and filtration on a 0.45 μm filter. Amounts of soluble anions, in terms of milligrams of anion per grams of LW and weight percentage with respect to the weight of removed patina, were reported in Table 1.

**Table 1:** Amounts of soluble anions detected on degraded samples as mean values with their standard errors

Anion	mg/g*	%**
HCO_2_^−^	0.044 ± 0.002	1.99 ± 0.02
CH_3_CO_2_^−^	0.125 ± 0.004	5.6 ± 0.2
Cl^−^	0.004 ± 0.001	0.17 ± 0.01
NO_3_^−^	0.006 ± 0.001	0.28 ± 0.01

*respect to the total weight of lead samples; **respect to the weight of removed patina

Acetate results to be the most abundant anion (5.6 ± 0.2 %), followed by formate (1.99 ± 0.02 %), nitrate (0.28 ± 0.01) and chloride (0.17 ± 0.01). These results are in line with the ambient exposure of these LW to acid vapours. As a consequence, the release of VOCs, especially AcOH and FOH, from the wood of the piano plays an important role in the degradation of its LW. In more detail, the piano under study is made of Bavarian spruce, which, as softwood, releases elevated concentrations of AcOH and FOH, prompting the formation of lead acetate and formate on LW surface (Adamová et al. 2019). Among all the degradation compounds, we can clearly observe the signals of hydrocerussite from XRD and FTIR spectra, since this compound is the main component of degraded patina. If, on the one hand, the detection of a not negligible amount of acetate and formate confirms the exposure of lead to organic acids, it also pushes toward the search for the contribution of these acids in accelerating the degradation of these LW and promoting the formation of hydrocerussite.

### Samples exposed to formic and acetic acid vapours

**Fig. 3:**
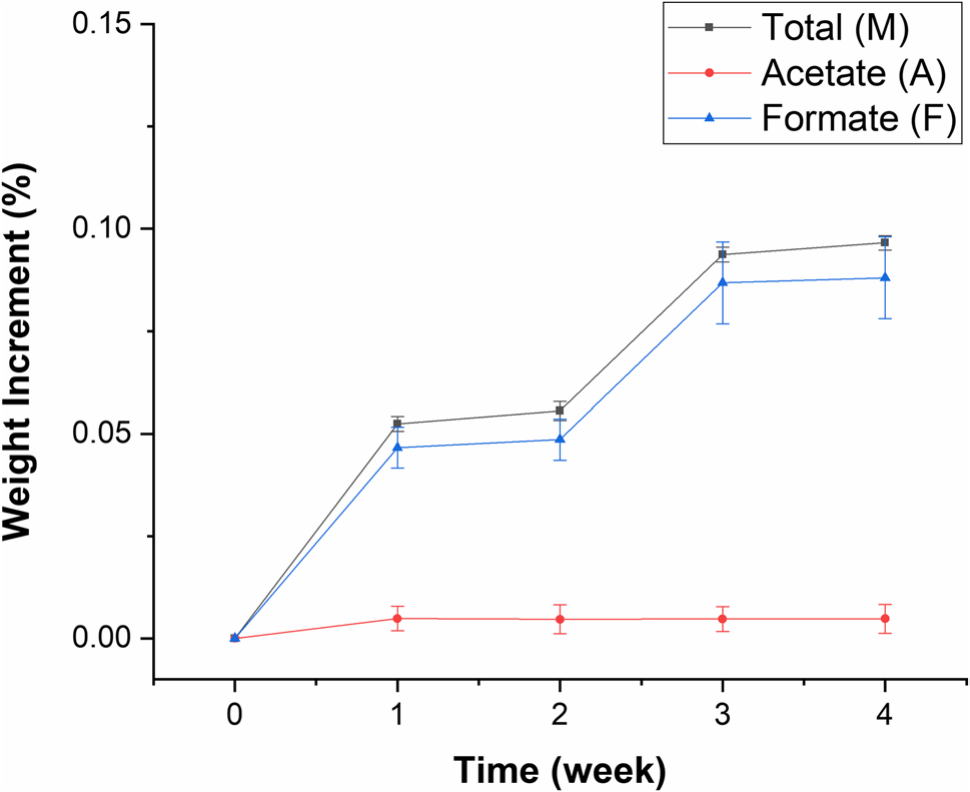
Weight increment (%) of the total mass M (■), mass of acetate A (  ) and mass of formate F (  ) with their standard errors

In order to evaluate the effect of both FOH and AcOH on LW deterioration, undegraded samples were exposed to FOH and AcOH vapours in controlled conditions: RH around 54%, close to that typical of museum environments, and FOH and AcOH concentration of 246 ± 25 mg/m^3^ and 115 ± 12 mg/m^3^, respectively. Anionic chromatography on the soluble part of the formed patina, allowed us to detect mainly formate and small amounts of acetate. For each exposure time (1, 2, 3, 4 weeks), the total weight increment (M) and that related to acetate (A) and formate (F) were calculated according to Eqs. 1, 2 and 3. Figure 3 shows the trend of calculated values as a function of exposure time.

The total weight increment (M) and the formate weight increment (F) follow the same trend with the increase of exposure time: a large rise in the first week, which then holds constant in the second week, then increases again in the third week and remains constant in the fourth week. On the contrary, acetate has a weight increment (A) that remains almost constant as exposure time changes. The higher amount of formate detected with respect to acetate (up to nearly 10 times higher) could be due to the fact that the action of AcOH on lead can be hindered in the presence of a comparable amount of FOH (Tétreault et al. 2003). The weight increment rate changes of the formic acid were probably due to an initial fast rate of formate formation on the bare lead and then possibly due to changes in the rate of formate formation associated with its conversion to hydrocerussite.

Although many studies have looked at lead corrosion triggered by exposure to AcOH and FOH alone (Niklasson et al. 2005, 2008; Gibson and Watt 2010; Malagodi et al. 2017), only one example involves the simultaneous presence of AcOH and FOH (Niklasson et al. 2007). In the latter case, a synergistic effect was observed due to the simultaneous presence of the two acids: corrosion is faster and more difficult to remove. Moreover, the predominant formation of a water-soluble crystalline phase (unassigned structure) containing both formate and acetate was observed (Niklasson et al. 2007).

**Fig. 4:**
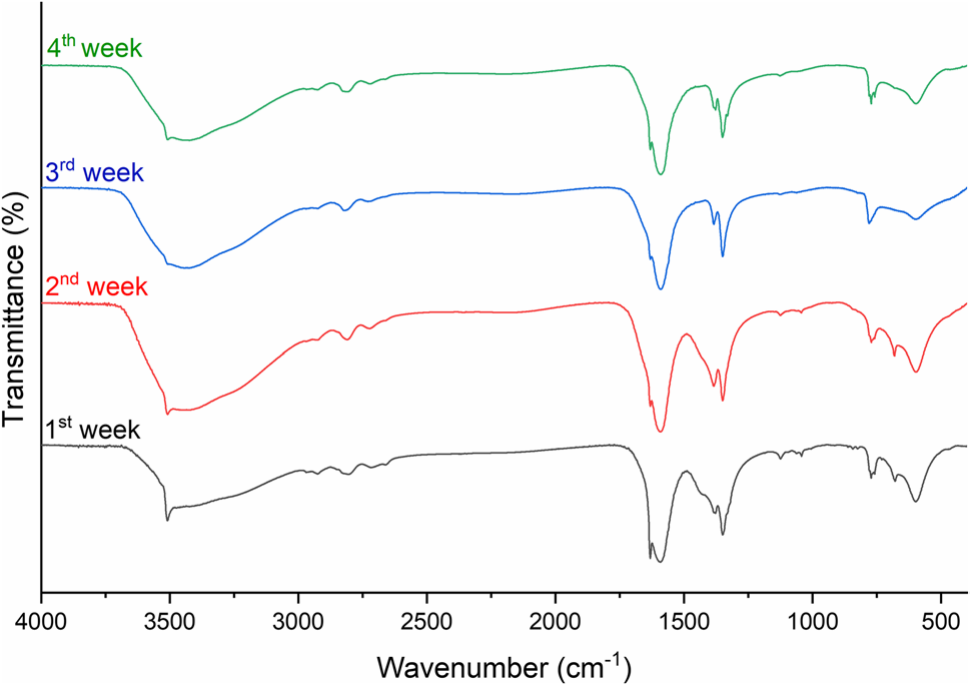
FTIR spectra of degradation compounds formed after 1 week (black), 2 weeks (red), 3 weeks (blue) and 4 weeks (green) of exposure to FOH and AcOH vapours

**Fig. 5:**
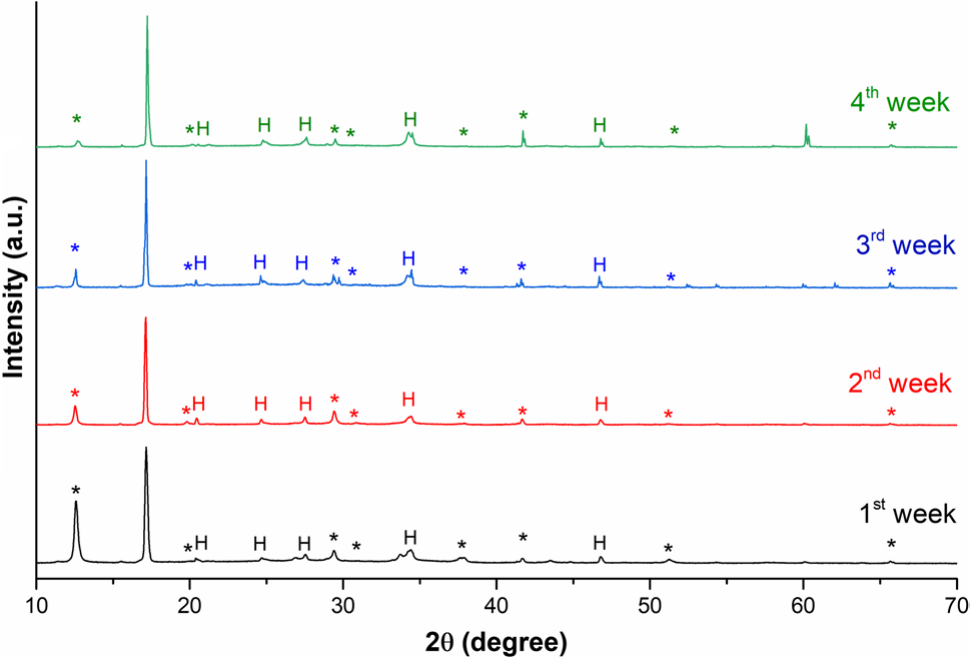
XRD spectra of degradation compounds formed after 1 week (black), 2 weeks (red), 3 weeks (blue) and 4 weeks (green) of exposure to FOH and AcOH vapours; *basic lead formate; H, hydrocerussite

In this study, to find the chemical nature of degradation compounds formed under controlled exposure to acid vapours, FTIR (Fig. 4) and XRD (Fig. 5) measurements were performed on the patina removed by sonication in water at different exposure times.

FTIR spectra show the typical signal of carboxylates: C=O stretching at 1590 cm^−1^ and 1628 cm^−1^, C-O stretching at 1346 cm^−1^ and 1385 cm^−1^. As exposure time increases from 1 week to 4 weeks, there is a reduction in the intensities of the bands at 1385 cm^−1^ and 1628 cm^-1^.

X-ray diffraction spectra of samples exposed to FOH and AcOH vapours (Fig. 5) show the diffraction pattern of both basic lead formate (*), Pb_2_O(HCOO)_2_ and hydrocerussite (H). Peaks with 2θ (hkl) 12.5° (110), 19.8° (111), 29.4° (221), 30.7° (002), 38° (330), 41.5° (241), 51.2° (350) and 65.6° (114) were assigned to the orthorhombic (Cccm) structure of basic lead formate (Mauck et al. 2010). At the same time, identified hydrocerussite peaks are at 2θ (hkl) 20.4° (101), 24.6° (104), 27.4° (015), 34° (009) and 47° (027).

As the exposure time increases, the intensity of basic lead formate peaks decreases slightly with a corresponding increase in the intensity of hydrocerussite peaks. Regarding the FTIR spectra (Fig. 4), it is likely that the hydrocerussite signals are masked by the more intense signals of lead carboxylates. No signals attributable to crystalline structures of lead acetates are observed. This may be due, in the first instance, to the lower measured amount of acetate compared to formate. In addition, a very intense signal at 17.2° is observed, which could be associated with crystalline forms containing both lead formate and acetate, as previously reported (Niklasson et al. 2007).

The results of our study confirm how exposure to AcOH and FOH vapours, under controlled humidity conditions, promotes lead corrosion and hydrocerussite formation over time, even in the case of piano LW. The effect of these acids on the atmospheric corrosion of lead suggests that it is important to monitor the concentration of both AcOH and FOH in theatres and museums.

## Conclusions

In this paper, the degradation compounds on LW from a *Steinway & Sons* piano were characterized using a multi-analytical approach. XRD and FTIR measurements allowed to identify hydrocerussite as the main component of the degradation products, whereas acetate (5.6 ± 0.2%) and formate (1.99 ± 0.02 %) are the most abundant among soluble anions detectable by IC analyses. Accelerated corrosion experiments in closed environments highlight how exposure of LW to AcOH and FOH vapours leads to the prevalent formation of basic lead formate, which promotes the formation of hydrocerussite over time. These results proved that VOCs (AcOH and FOH) emitted from the wood of the piano may trigger the degradation of piano LW.

### Supplementary information

ESM 1 (DOCX 24 kb)

## Data Availability

Not applicable
