# Supplementary material for: Insight on the deterioration of cultural objects: a multi-analytical approach to characterize degradation products of lead weights from a Steinway & sons piano
Source: Environ Sci Pollut Res Int. 2023 Sep 14;30(47):104633–9. doi: 10.1007/s11356-023-29790-1 (PMC10567956; doi:10.1007/s11356-023-29790-1)
Supplement: Supplementary file 1 — (DOCX 24 kb) [file 11356_2023_29790_MOESM1_ESM.docx]

**Supporting Material**

**Insight on the deterioration of cultural objects: a multi-analytical approach to characterize degradation products of *lead weights* from a *Steinway & sons* piano**

Antonio Faggiano^a,b,1^, Concetta Pironti^b,c,1^, Oriana Motta^b,c^, Ylenia Miele^a^, Antonino Fiorentino^a,b^, Nadia Marchettini^d^, Maria Ricciardi^a,b*^, Antonio Proto^a,b^

^a^Department of Chemistry and Biology, University of Salerno, via Giovanni Paolo II 132, 84084 Fisciano (SA), Italy

^b^Consorzio Interuniversitario per la Scienza e la Tecnologia dei Materiali (INSTM), 50121 Firenze, Italy

^c^Department of Medicine Surgery and Dentistry, University of Salerno, via S. Allende, 84081 Baronissi (SA), Italy

^d^Department of Earth, Environmental and Physical Sciences, University of Siena, Pian dei Mantellini 44, 53100 Siena, Italy

^1^these authors contribute equally to this work

*Corresponding author: Ricciardi Maria, University of Salerno, via Giovanni Paolo II 132, 84084 Fisciano (SA), Italy, tel +39 89968175 [mricciardi@unisa.it](mailto:mricciardi@unisa.it)

**Summary**

[**Determination of air concentration of formic and acetic acid** 3](#_Toc131182472)

[**Ion chromatography (IC)** 3](#_Toc131182473)

[**X-ray diffraction (XRD) analyses** 3](#_Toc131182474)

[**Fourier Transform-Infrared (FT-IR) spectroscopy** 3](#_Toc131182475)

## **Determination of air concentration of formic and acetic acid**

The concentration (C, mg/m^3^) of acids in the vapour phase were calculated using the Equation (S1):

$$C= {(m*l)}/{(S*D*t)} (S1)$$

where *m* is the mass (mg) of acetic or formic acid, *l* is the diffusion length (0.071 m), *S* is the cross-sectional area of the tube (9.5 x 10^-5^ m^2^), *D* is the diffusion rate of acetic or formic acid in air (1.1 x 10^-5^ or 1.27 x 10^-5^ m^2^ s^-1^, respectively), and *t* is the time of exposure (s).

## **Ion chromatography (IC)**

Ion-exchange chromatography analyses of water extracts were performed using a Thermo Scientific-DionexTM AquionTM ion chromatograph equipped with a conductivity system detector, a self-regenerating suppressor and A Dionex IonPac AS20 hydroxide selective anion-exchange column. Ionic concentrations (expressed as mg/L) were obtained using calibration curves prepared employing CH_3_CO_2_Na, HCO_2_Na, NaNO_3_, NaCl, and Na_2_SO_4_ to prepare standard anion solution in the range 0.3-20 mg/L. Limit of detection (LOD) and limit of quantification (LOQ) were calculated based on the standard deviation of the response and the slope, using the following equations:

$$LOD={(3.3* \sigma)}/S (S2)$$

$$LOQ={(10* \sigma)}/S (S3)$$

where *σ* is the standard deviation of the calibration curve and *S* is the slope of the calibration curve.

## **X-ray diffraction (XRD) analyses**

X-ray diffraction analyses were carried out with Bruker D2 Advance automatic diffractometer operating with a nickel-filtered CuKα radiation, recording data in the 2θ range: 4-80° with the resolution of 0.02°. XRD spectra analysis was done using Match! Software with the support of COD (Crystallography Open Database) library.

## **Fourier Transform-Infrared (FT-IR) spectroscopy**

FTIR spectra were collected with a Vertex70 spectrometer (Bruker). The measurements were carried out in transmission mode (range 500-4000 cm^−1^, resolution of 4 cm^−1^) on a potassium bromide disk prepared by mixing the powdered sample with FTIR-grade KBr.
